# The Leishmania major BBSome subunit BBS1 is essential for parasite virulence in the mammalian host

**DOI:** 10.1111/mmi.12383

**Published:** 2013-09-17

**Authors:** Helen P Price, Daniel Paape, Michael R Hodgkinson, Katie Farrant, Johannes Doehl, Meg Stark, Deborah F Smith

**Affiliations:** 1 Centre for Immunology and Infection Department of Biology, University of YorkHeslington, York, YO10 5YW, UK; 2 Technology Facility Department of Biology, University of YorkHeslington, York, YO10 5YW, UK

## Abstract

Bardet–Biedl syndrome (BBS) is a human genetic disorder with a spectrum of symptoms caused by primary cilium dysfunction. The disease is caused by mutations in one of at least 17 identified genes, of which seven encode subunits of the BBSome, a protein complex required for specific trafficking events to and from the primary cilium. The molecular mechanisms associated with BBSome function remain to be fully elucidated. Here, we generated null and complemented mutants of the BBSome subunit *BBS1* in the protozoan parasite, *Leishmania*. In the absence of BBS1, extracellular parasites have no apparent defects in growth, flagellum assembly, motility or differentiation *in vitro* but there is accumulation of vacuole-like structures close to the flagellar pocket. Infectivity of these parasites for macrophages *in vitro* is reduced compared with wild-type controls but the null parasites retain the ability to differentiate to the intracellular amastigote stage. However, infectivity of *BBS1* null parasites is severely compromised in a BALB/c mouse footpad model. We hypothesize that the absence of *BBS1* in *Leishmania* leads to defects in specific trafficking events that affect parasite persistence in the host. This is the first report of an association between the BBSome complex and pathogen infectivity.

## Introduction

Bardet–Biedl syndrome (BBS) is a rare autosomal recessive disorder in humans characterized by primary cilium dysfunction (Forsythe and Beales, 2013). Mutations in 17 different genes have been implicated in this condition, many of which are restricted to ciliated and flagellated species (Chiang *et al*., 2004; Fan *et al*., 2004; Hodges *et al*., 2010; Forsythe and Beales, 2013). Seven of these genes encode subunits which (together with BBIP10) assemble into an octomeric complex termed the BBSome (Nachury *et al*., 2007). Evidence from animal models of BBS indicates that the BBSome is involved in specific transport events to and from the cilium but is not required for cilium assembly in most cell types (Mykytyn *et al*., 2004; Lechtreck *et al*., 2009). The BBSome in mice is required for cilium localization of the G-protein coupled receptors (GPCR) somatostatin receptor 3 and melanin-concentrating hormone receptor 1 in hippocampal neurones (Berbari *et al*., 2008) and for export of the GPCR dopamine receptor 1 from neuronal cilia (Domire *et al*., 2010). Disruption of Bbs1, Bbs4 or Bbs7 protein function in *Chlamydomonas reinhardtii* disrupts phototaxis due to a defect in export of signalling proteins including phospholipase D from the cilium (Lechtreck *et al*., 2009; 2013[Bibr b36],[Bibr b35]).

Although the molecular pathways governed by the BBSome are still under investigation, it is clear that the subunit BBS1 plays a central role in effector binding to the complex (Nachury *et al*., 2007; Jin *et al*., 2010). In a study characterizing the sequential assembly process of the BBSome, BBS1 and BBS4 were identified as the final subunits to be incorporated into the complex (Zhang *et al*., 2012). Most recently, the BBS1 orthologue in *Caenorhabditis elegans* (BBS-1) was identified in a whole-genome mutagenesis screen as an important mediator of intraflagellar transport (IFT) particle assembly at the base of the cilium and of IFT turnaround upon arrival at the ciliary tip (Wei *et al*., 2012). BBS-1 is able to bind to the *C. elegans* IFT structural protein DYF-2 (human WDR19/*Chlamydomonas* IFT144) which was also identified in the IFT mutagenesis screen and this interaction is believed to link the BBSome with the IFT machinery (Wei *et al*., 2012). In humans, there is a direct association between BBS1 and the Rab8 guanine nucleotide exchange factor (GEF) Rabin8 (Nachury *et al*., 2007), while BBS1 can also bind to the small GTPase ARL6 which recruits the complex to the primary cilium (Jin *et al*., 2010).

No studies have been reported to date on the effects of depleting BBSome subunits in flagellated protozoa. However, we previously reported that ARL6, a binding partner of BBS1, is found on small vesicles throughout the body of the protozoan parasite *Trypanosoma brucei* (Price *et al*., 2012). Knock-down of the expression of *T. brucei* ARL6 causes a significant decrease in flagellum length but this does not have detrimental effects on motility or infection in an experimental mouse model. Further, overexpression of BBS1 in *T. brucei* results in the translocation of ARL6 to the flagellar pocket, suggesting a conserved functional link between BBS1 and ARL6 across the ciliated/flagellated eukaryotes (Price *et al*., 2012).

Here we describe studies on BBS1 in the related protozoan *Leishmania major*, one of the causative agents of leishmaniasis, a spectrum of neglected tropical diseases that affect 12 million people and threaten 350 million worldwide (Alvar *et al*., 2012). *L. major* has a digenetic life cycle with a promastigote stage residing inside the midgut of the sand fly vector *Phlebotomus papatasi* and an obligate intracellular amastigote stage found in phagolysosomal-like parasitophorous vacuoles within host macrophages (Herwaldt, 1999). The promastigote stage has a single motile flagellum with microtubule pairs arranged in a 9 + 2 configuration and a kinetoplastid-specific extra-axonemal structure termed the paraflagellar rod (PFR) (Vickerman, 1962; Gibbons, 1981). The promastigote flagellum is important for migration through the peritrophic matrix (that surrounds the bloodmeal) to the sand fly midgut and for subsequent attachment to the midgut epithelium via surface glycoconjugates, a vital step in the establishment of infection (Warburg *et al*., 1989; Pimenta *et al*., 1994; Bates, 2008). The flagellum also has a role in transmission of metacyclic promastigotes from the sand fly to the mammalian host. A live imaging study using *Leishmania donovani* showed that a majority of parasites attach to the macrophage surface by the flagellum (particularly the flagellum tip) triggering actin-dependent phagocytosis (Forestier *et al*., 2011). The metacyclic promastigote then differentiates into the amastigote stage which has a very short immotile flagellum of unknown function with a 9 + 0 microtubule pair configuration similar to that of primary cilia (Alexander, 1978; Gluenz *et al*., 2010).

Our data presented here demonstrate that *L. major* parasites that are null for *BBS1* show normal growth, flagellum assembly and motility in the promastigote form *in vitro*. Loss of *BBS1* does not prevent the infection of macrophages by metacyclic promastigotes or differentiation into intracellular amastigotes but *BBS1* null parasites are unable to persist or induce production of a lesion in a mouse footpad model of infection. Thus, subunit BBS1 of the BBSome complex, which is widely associated with cilium function, appears to be most important in *Leishmania* parasites at the immotile amastigote stage. Our findings suggest either that the tiny amastigote flagellum has an essential BBSome-dependent signalling or sensing role in the host environment or that the functions of the BBSome are not restricted to flagellar trafficking in these organisms. This is the first report linking BBSome function to pathogen virulence to date.

## Results and discussion

### BBS1 is transcribed throughout the L. major life cycle

Genomes of the kinetoplastid parasites code for divergent orthologues of all eight subunits of the BBSome complex, with a range of 25–44% identity between human and *L. major* sequences at the amino acid level. The *L. major* orthologue of BBS1 (LmjF.35.4180) encodes a 64 kDa protein which shares 31% identity with human BBS1 and both proteins contain a putative WD40 repeat region (residues 22–388 of 592 in *L. major*) predicted to form a seven-bladed β-propeller.

To test the expression profile of *BBS1* during progression through the *L. major* life cycle, quantitative RT-PCR was performed on total RNA extracted from *L. major* promastigotes grown in culture for 2 days (procyclic) and 7 days (metacyclic) and from amastigotes extracted from the lymph node draining the footpad of a BALB/c mouse infected with wild-type *L. major* for 6 weeks (see Supplementary Fig. S1A). No significant differences were found in the level of *BBS1*-specific transcript in the three life cycle stages. Protein levels could not be tested as no specific antibody is yet available for *Leishmania* BBS1.

### BBS1 is not essential for growth of L. major promastigotes in vitro

**Figure 1:**
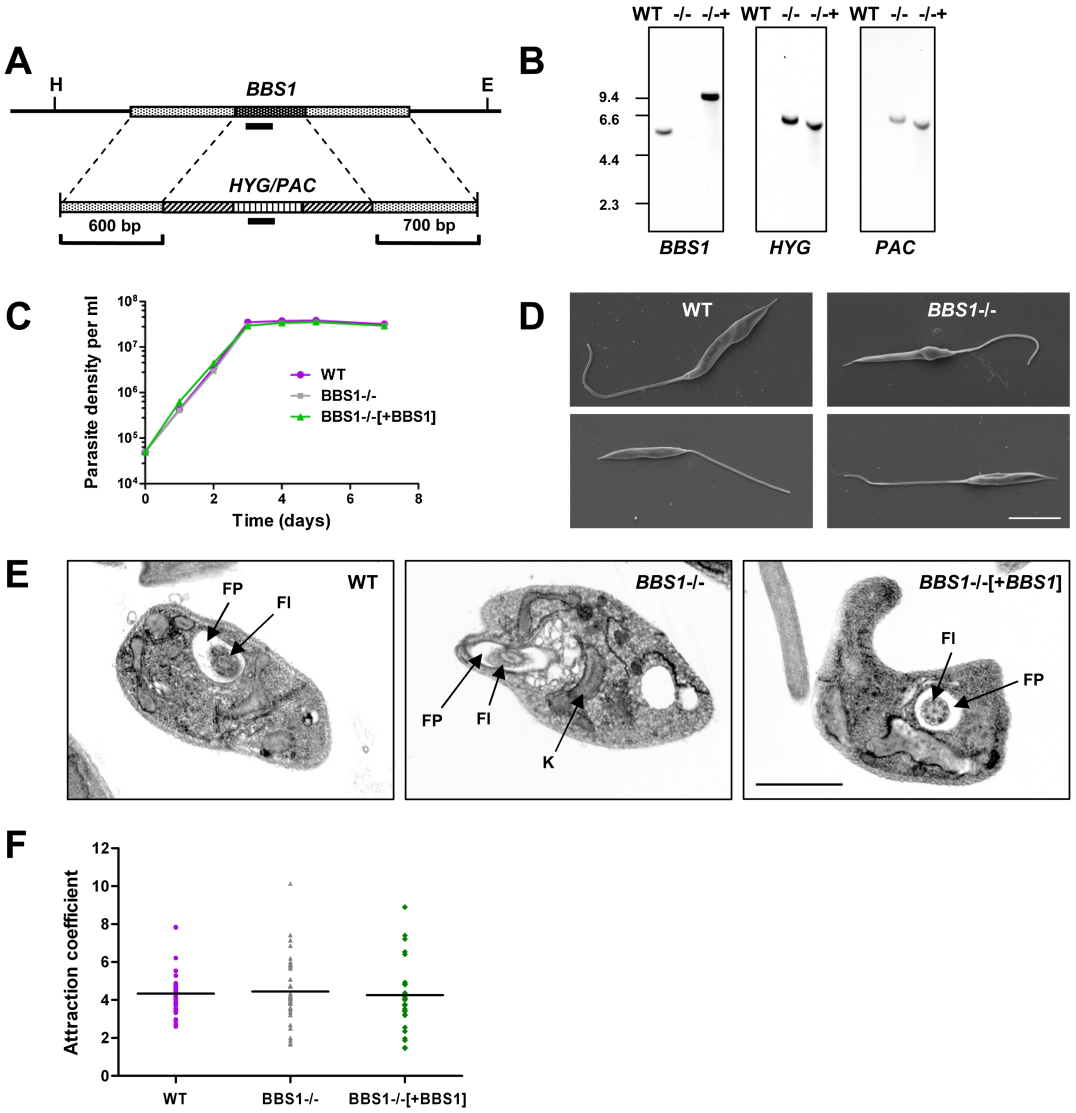
*BBS1* gene deletion in *Leishmania major*.A. Schematic diagram of the *LmBBS1* locus and the plasmid constructs used for targeted deletion of the locus by replacement with hygromycin/puromycin resistance genes (*HYG*/*PAC*). Flanking sequences used to generate the targeting vectors are shown. Solid black bars represent fragments used as hybridization probes. H, HindIII site. E, EcoRV site.B. Southern blot analysis of wild-type *L. major* (WT), *BBS1* null (−/−) and complemented (−/−+) parasite lines. Five micrograms of genomic DNA from each parasite line was digested with HindIII/EcoRV, size separated through 0.8% agarose, blotted and hybridized with DIG-labelled DNA probes (∼ 200 bp) as indicated. Corresponding DNA marker positions are shown (Kb).C. Growth of promastigotes from wild-type *L. major* (WT), *BBS1* null (BBS1−/−) and complemented (BBS1−/−[+BBS1]) monitored over a 7-day time-course. Mean values are shown (*n* = 3) ± SD (error bars are not visible).D. Scanning electron micrographs of *L. major* wild-type and *BBS1* null cell lines as above. Bar, 5 μm.E. Transmission electron micrographs of promastigotes from *L. major* parasite lines as above. FP, flagellar pocket. Fl, flagellum. K, kinetoplast. Bar, 2 μm.F. Procyclic promastigote osmotaxis assay. Parasite lines as above (day 3 post-inoculation) were tested for their ability to migrate into capillary tubes containing agarose and 100 mM sucrose compared with movement into control capillary tubes containing agarose alone (*n* = 6).Data shown are the combined results of six independent experiments.

In order to characterize the function of BBS1 in *L. major*, both alleles of the gene were replaced with antibiotic resistance genes *HYG* and *PAC* to produce double knockout lines (ΔBBS1::HYG/ΔBBS1::PAC), as illustrated in Fig. 1A. Complemented lines were also produced in which a single copy of the *BBS1* open reading frame with a tdTomato N-terminal tag was integrated into the genome of a double knockout line at a single site within the tandemly repeated rRNA loci (ΔBBS1::HYG/ΔBBS1::PAC [NEO TdTomato BBS1]). qPCR on genomic DNA from selected complemented lines showed that one copy of the gene had been inserted into the rRNA locus (data not shown). However, q-RT-PCR demonstrated a 14-fold increase in *BBS1-*specific transcript in procyclic promastigotes of one of these complemented lines compared with wild-type cells (Supplementary Fig. S1B), indicating overexpression of *BBS1*. Localization of the TdTomato-tagged BBS1 protein was investigated by fluorescence imaging. As the fluorescent signal was relatively weak, cells were also stained with an anti-dsRed antibody. In both cases, the BBS1 protein was detected throughout the cell body, was found at higher levels in several bright foci between the kinetoplast and nucleus and close to the base of the flagellum, and was excluded from the nucleus (Supplementary Fig. S1C and D). We could not exclude the possibility that the large fluorescent tag was interfering with the localization of this protein, therefore we also generated a parasite line expressing LmBBS1 with a C-terminal V5 epitope tag. Immunofluorescence analysis using an anti-V5 antibody showed localization of this protein was very similar to that of the TdTomato-tagged form (Supplementary Fig. S1E). Concentration of the protein in several bright foci was evident between the kinetoplast and nucleus, a location that may represent part of the endosomal system.

Southern hybridization was used to confirm the correct integration of exogenous DNA into the *L. major* genome. HindIII/EcoRV-digested genomic DNA hybridized with a *BBS1* ORF probe (Fig. 1B, first panel) revealed a band of 5 Kb in wild-type *L. major*. This band was not seen in double knockout or complemented lines but the latter had a band of 9.5 Kb corresponding to the inserted copy of the gene at the rRNA locus. *HYG* and *PAC* probes (Fig. 1B, second and third panels) produced single bands of 6 Kb in double knockout and complemented cell lines but no signal in wild-type cells as expected. Cell growth of the transgenic lines was monitored over 7 days and no significant differences were observed between the parental line and the *BBS1* mutant lines (Fig. 1C). Therefore, BBS1 is not required for normal growth of *L. major* promastigotes in culture. This correlates with studies in other eukaryotic organisms, including mice, zebrafish, *C. elegans* and *C. reinhardtii*, in which the BBSome complex subunits are not required for viability (Mykytyn *et al*., 2004; Nishimura *et al*., 2004; Zhang *et al*., 2013).

### Loss of BBS1 causes morphological changes in the flagellar pocket in L. major promastigotes

Gross morphology was unaffected by the loss of *BBS1* and flagellum assembly appeared normal in the knockout line at the early promastigote stage as shown by scanning electron microscopy and immunofluorescence (Fig. 1D, Supplementary Fig. S1F and G and Supplementary Fig. S2A respectively). Flagellum and cell body lengths (day 4 of culture) were not significantly different between the BBS1 null and wild-type cell lines (Supplementary Fig. S1F and G) although the mean body length was significantly greater in the BBS1 complemented line compared with wild-type (*P* value < 0.001). Correlating with normal flagellum assembly, no defects in parasite motility were observed in culture in the BBS1 mutant lines (data not shown). This agrees with previous studies in other eukaryotic species, in which disruption or deletion of BBSome subunits does not affect ciliogenesis, with the exception of mammalian spermatozoa which lack motile flagella in BBS knockout mice (Mykytyn *et al*., 2004; Nishimura *et al*., 2004; Zhang *et al*., 2013). However, transmission electron microscopy revealed an accumulation of vacuole-like structures in the *L. major BBS1* null line in the region surrounding the flagellar pocket, the sole site of endo- and exocytosis (Fig. 1E and Supplementary Fig. S2B–D). These vacuoles were observed in approximately 50% of the *BBS1* null cell sections in which the flagellar pocket was visible (*n* = 60), but were not seen in either wild-type or *BBS1* complemented cells. These may be indicative of a defect in membrane trafficking at this site or could be a secondary effect due to other physical changes, e.g. redistribution of lipids or ion imbalance. This phenotype differs from that described in *Chlamydomonas*, in which ciliogenesis proceeds as normal in BBSome mutant lines but there is accumulation of vesicles containing signalling proteins inside the cilium due to a defect in retrograde transport. A 150-fold enrichment in phospholipase D has been shown in the cilia of BBS4 mutant *C. reinhardtii* cells which in turn causes changes in lipid composition (Lechtreck *et al*., 2009; 2013). Unlike *C. reinhardtii*, *L. major* does not disassemble its flagellum upon mitosis and therefore any accumulation of proteins would be expected to become progressively worse with time in this latter species. However, our data show no evidence of vesicles within the *L. major BBS1* null flagellum (Fig. 1E and Supplementary Fig. S2B–D).

### Loss of BBS1 does not affect fluid-phase endocytosis of FM4-64

Fluid-phase endocytosis was tested by incubation of procyclic promastigotes in the lipophilic styryl dye FM4-64 (Supplementary Fig. S2E and F). In all three parasite lines, FM4-64 was initially detected predominantly in the flagellar pocket. By 40 min post-incubation, there was evidence in all cell lines of trafficking to another dense compartment, identified by counter-staining to be the lysosome (data not shown). Trafficking of this compound was quantified by scoring parasites (250 per sample) for the presence of fluorescence in the flagellar pocket, in multiple foci around the pocket (endosomes) and at a single focus between the kinetoplast and the nucleus (lysosome) (Supplementary Fig. S2E). No differences were seen between wild-type and *BBS1* mutant cell lines, allowing us to conclude that the absence of *BBS1* does not appear to affect fluid-phase trafficking of FM4-64 in this life cycle stage.

### BBS1 is not required for osmotaxis in L. major promastigotes

We tested the ability of promastigotes from the BBS1 mutant lines to migrate towards an attractant (sucrose) in glass capillary tubes, as described previously (Leslie *et al*., 2002). This is an osmotaxis response, which may be important for navigation of the parasite in the gut of the sand fly vector. Osmotaxis in *L. major* has been linked to aquaglyceroporin which localizes to the flagellum in promastigotes (Figarella *et al*., 2007). A defect in trafficking of this pore-family protein could potentially affect the osmotaxis response in affected parasites. However, our results (Fig. 1F) show no significant differences in attraction to sucrose between wild-type and BBS1 mutant lines, indicating that flagellar proteins involved in this process are being trafficked normally in the promastigote.

### Attempts to visualize the IFT system in L. major

As the *C. elegans* BBS-1 protein has recently been reported to interact directly with the IFT structural protein DYF-2 (IFT144) (Wei *et al*., 2012), attempts were made to visualize IFT particles in *L. major* promastigotes as previously described in *T. brucei* (Buisson *et al*., 2013). A series of transgenic *L. major* lines were generated on both wild-type and *BBS1* null backgrounds, expressing the parasite orthologues of IFT52 and IFT27 with C-terminal GFP tags. Fluorescence imaging of live immobilized cells showed that IFT52-GFP and IFT27-GFP localized predominantly to the base of the flagellum, cytosol and to a lesser extent to the extracellular region of the flagellum (Supplementary Fig. S3A and B), with no clear differences between wild-type and *BBS1* null lines. IFT particles were faintly labelled in a minority of cells but were not sufficiently distinct for accurate kinetic analysis. IFT52-GFP also localized to the cytosol in the intracellular amastigote stage, which has a tiny immotile flagellum (data not shown). As an alternative approach, cells were probed with an antibody against *T. brucei* IFT172. This stained both the parasite body and flagellum of procyclic promastigotes and no differences were seen between the three *L. major* lines (Supplementary Fig. S3C).

In summary, visualization of the IFT system in the flagella of *L. major* promastigotes using similar methods to those used in other systems has proved challenging. Further studies using specific antibodies against *L. major* IFT proteins are now required to resolve the technical issues encountered. However, it is interesting to note that less than half of the IFT protein pool in *T. brucei* has been predicted (by modelling) to be used in active flagellum transport, indicating that these proteins could also be involved in other cellular functions (Buisson *et al*., 2013). Our previous study showed that the BBSome-interacting small GTPase ARL6 is not localized to the flagellum as expected but is found on small vesicle-like structures throughout the parasite body (Price *et al*., 2012). If the IFT system has a broader function and distribution in kinetoplastids than in other organisms, it is conceivable that the BBSome in these parasites is similarly divergent.

### Proteomic analysis of flagellar axonemes in BBS1 mutant lines

Biochemical analysis was performed to identify any significant differences in flagellum composition in BBS1 mutants. Flagellar axoneme fractions were isolated from procyclic promastigotes by detergent/NaCl extraction as described previously for *T. brucei* (Broadhead *et al*., 2006). Analysis by 2D gel electrophoresis showed no significant differences in the protein composition of extracts from wild-type, *BBS1* null and complemented lines (Supplementary Fig. S4) within the detection limits of these methods (where the lower detection limit of Sypro Ruby is 1–2 ng protein). To confirm the flagellar composition of the detergent/NaCl extracts, 60 major spots were excised from representative gels and identified by MS/MS (see Supplementary Table S2). As expected, a large proportion of spots were found to contain α/β tubulin. Other known flagellar components were also identified, including paraflagellar rod proteins PFR1D and 2C, KMP11, the *N*-myristoylated protein SMP1, centrin and calmodulin, confirming the extracts to be largely composed of flagellar axonemes. Further work is required to optimize methods for the isolation of intact promastigote flagella and analyse the effects of BBS1 depletion on target flagellar membrane proteins.

### L. major  BBS1 is not required for metacyclogenesis in vitro

Metacyclogenesis, the differentiation of *Leishmania* parasites from the dividing procyclic to the non-dividing metacyclic promastigote stage, is known to be vital for host infectivity (da Silva and Sacks, 1987). Differentiation naturally occurs in the sand fly midgut in preparation for transmission into the host but can also be observed in culture during the stationary phase of the growth cycle (Sacks and Perkins, 1984). During this developmental transition, there is an increase in the ratio of flagellum length to cell body length (Sacks *et al*., 1985), a substantial increase in the size and complexity of the abundant surface glycoconjugate lipopolysaccharide (LPG) (Sacks *et al*., 1985; 1990[Bibr b52],[Bibr b51]) and upregulated expression of the metacyclic marker proteins HASPB and SHERP (Denny *et al*., 2000; Knuepfer *et al*., 2001; Sadlova *et al*., 2010).

**Figure 2:**
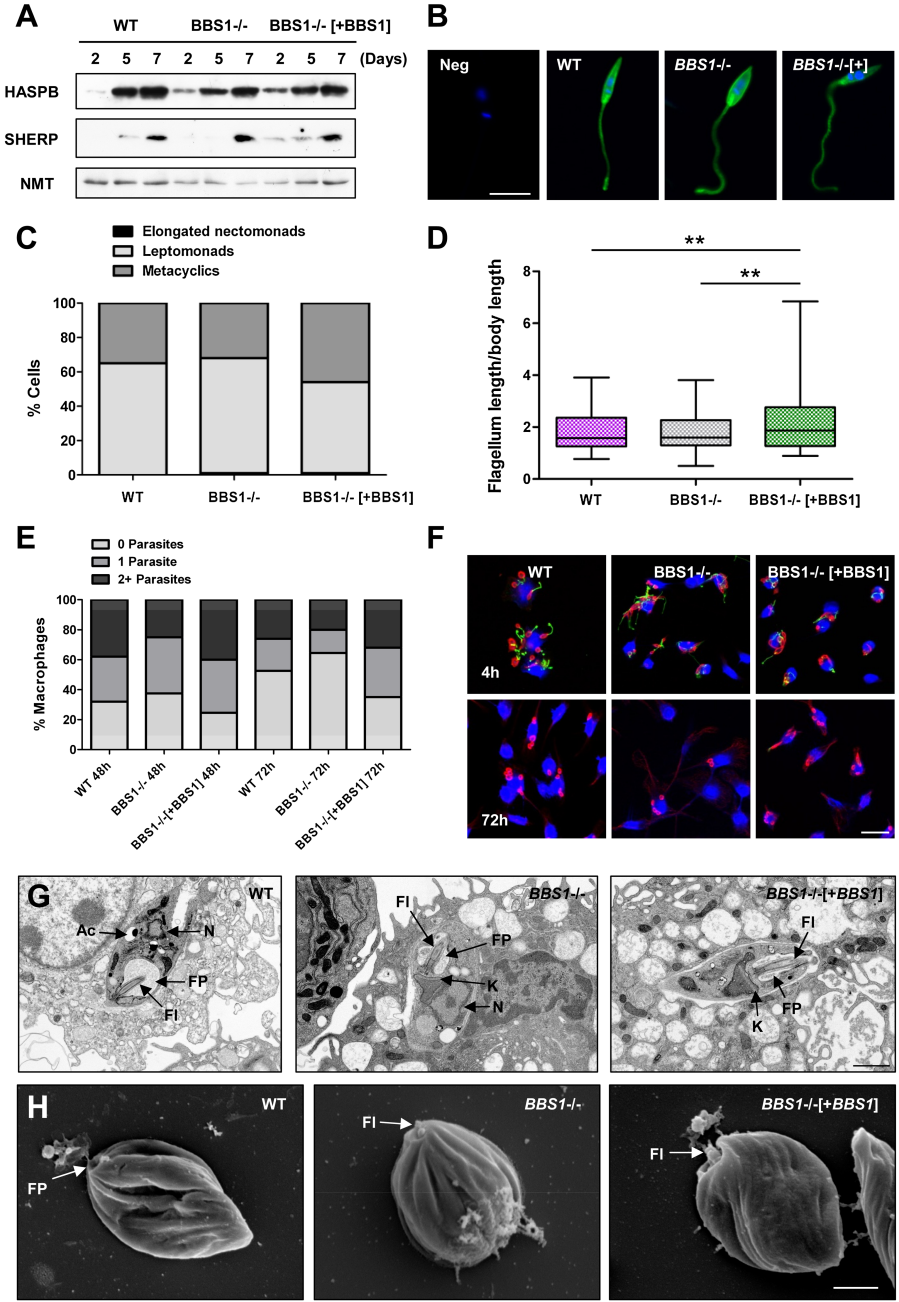
Effect of *BBS1* gene deletion on *L. major* differentiation and macrophage infection *in vitro*.A. Procyclic promastigotes of *L. major* wild-type (WT), *BBS1* null (BBS1−/−) and complemented (BBS1−/−[+BBS1]) lines were used to inoculate cultures at an initial concentration of 5 × 10^4^ ml^−1^ and samples were collected at 2, 5 and 7 days. Total-cell lysates (1 × 10^6^ cells per lane) were immunoblotted and probed with antibodies against *L. major* HASPB and SHERP, with NMT as a constitutively expressed control.B. Immunofluorescence analysis of *L. major* parasite lines as above, following growth in culture for 7 days to promote differentiation from procyclic to metacyclic promastigotes. Parasites were probed with rabbit anti-HASPB (green) and co-stained with DAPI (blue). Neg, negative control wild-type procyclic promastigote which shows no HASPB expression. Bar, 5 μm.C. *L. major* lines analysed in (A) were harvested at day 7 post-inoculation and stained with antibodies against α-tubulin and *L. major* PFR1. A number of cellular dimensions were measured (200 parasites per sample) and the life cycle stage assigned to each parasite based on the strict set of criteria used to define developmental stages within the sand fly vector (Walters, 1993; Cihakova and Volf, 1997). Data shown represent one of two independent experiments.D. Flagellum length measurements divided by body length are shown for *L. major* lines as described in (C).E. Mouse peritoneal macrophages were infected with metacyclic promastigotes from wild-type and *BBS1* mutant lines. The number of parasites per macrophage (200 per sample) was determined at 48 and 72 h post-infection by immunofluorescence using antibodies against α-tubulin and *L. major* PFR1, excluding extracellular PFR1-positive metacyclic parasites from the analysis. The percentage of macrophages with 0, 1 or ≥ 2 parasites is shown for each group. These data represent one of three independent experiments.F. Immunofluorescence of mouse peritoneal macrophages following infection with *L. major* metacyclic promastigotes for 4 h or 72 h (as described in E). Cells were probed with anti-α-tubulin (red) and anti-PFR1 (green) and co-stained with DAPI (blue). Bar, 20 μm.G. Transmission electron micrographs of mouse peritoneal macrophages infected with metacyclic promastigotes from *L. major* parasite lines as above for 72 h. Ac, acidocalcisome. N, nucleus. FP, flagellar pocket. Fl, flagellum. K, kinetoplast. Bar, 2 μm.H. Scanning electron micrographs of *L. major* amastigotes isolated from human monocytic cell line THP1 infected with metacyclic promastigotes from parasite lines as above for 72 h. FP, flagellar pocket. Fl, flagellum. Bar, 1 μm.

The ability of BBS1 mutant lines to undergo metacyclogenesis in culture was analysed using two methods. Expression of the metacyclic marker proteins, HASPB and SHERP, was tested by immunoblotting total parasite lysates taken at days 2–7 following parasite inoculation. There were no obvious differences between the *BBS1* null and complemented lines and wild-type cells, with clear upregulation of both HASPB and SHERP proteins by day 7 (Fig. 2A) as described previously (Sadlova *et al*., 2010). In correlation, HASPB-expressing parasites with relatively short bodies and long flagella characteristic of metacyclic promastigotes could be seen by immunofluorescence at day 7 in all cases (Fig. 2B). Parasites at day 7 were also stained with antibodies against α-tubulin and *L. major* PFR1 and a number of cellular dimensions were measured, assigning a life cycle stage to each parasite based on a strict set of criteria that are used to define developmental stages within the sand fly vector (Walters, 1993; Cihakova and Volf, 1997). Analysis revealed no significant difference in the proportion of cells defined by this method as metacyclic promastigotes (flagellum length ≥ 2 × body length, body width < 4 μm) in wild-type and *BBS1* null lines (35% and 32% respectively, *P* value > 0.05). Therefore, the absence of BBS1 does not inhibit metacyclogenesis *in vitro*. In comparison, 46% of the *BBS1* complemented line were defined as metacyclic, which is significantly different from both wild-type and *BBS1* null lines (*P* value < 0.01) (Fig. 2C). It is interesting to note that parasites from the *BBS1* complemented line at day 7 had no difference in body length but had significantly longer flagella (*P* value < 0.001) compared with wild-type cells (Supplementary Fig. S5A and B). In correlation, the complemented line also had a significant increase in the ratio of flagellum length/body length (Fig. 2D) compared with wild-type and *BBS1* null lines (*P* value < 0.01), with the presence of parasites with a flagellum length up to seven times longer than the body. This may be the result of BBS1 overexpression (due to insertion of the replacement gene downstream of the rRNA promoter). However, the reverse effect is not seen in *BBS1* null parasites, which also showed a slight but significant increase in flagellum length (Supplementary Fig. S5B) but not a significant increase in the number of parasites classified as metacyclic promastigotes at day 7 (Fig. 2C).

Flagellum length in the green alga *Chlamydomonas* is controlled by an active process requiring an initial phase of rapid growth followed by a steady state of balanced assembly and disassembly once a defined length has been reached (Marshall and Rosenbaum, 2001; Song and Dentler, 2001; Marshall *et al*., 2005). The balance-point model predicts that the rate of assembly has an inverse relationship with flagellum length assuming that the number of IFT particles is fixed. In contrast, the rate of disassembly is independent of length and the two rates can only balance at a specific flagellum length (Marshall *et al*., 2005). Applying this model to our current data, an increase in flagellum length in metacyclic promastigotes of *BBS1* complemented lines could result from a reduced rate of disassembly and/or increased efficiency of assembly due to a faster rate of IFT movement, a greater number of particles or more cargo per particle.

### L. major  BBS1 influences but is not essential for macrophage infection in vitro

Parasite infectivity was analysed *in vitro* by infecting mouse peritoneal macrophages with metacyclic promastigotes from wild-type and BBS1 mutant lines. The number of parasites per macrophage was determined at regular time points by immunofluorescence using antibodies against α-tubulin and *L. major* PFR1, excluding extracellular PFR1-positive metacyclic parasites from the analysis. At 72 h, there were significantly more uninfected macrophages in the *BBS1* null group compared with infection with wild-type *L. major* (64.5% and 52.5% respectively, *P* value < 0.01) whereas there were significantly fewer uninfected cells for the *BBS1* complemented sample compared with the wild-type at this time point (35% and 52.5% respectively, *P* value < 0.01) (Fig. 2E and F). In addition, there were significantly more macrophages infected with 2 or more parasites for the *BBS1* complemented sample than for the *BBS1* null line, both at 48 h and at 72 h post-infection (*P* value < 0.001) (Fig. 2E). However, immunofluorescence analysis showed no significant differences in the ability of the three parasite lines to attach to macrophages within the first 4 h of incubation (Supplementary Fig. S5C). At 72 h post-infection round PFR1-negative parasites characteristic of intracellular amastigotes could be seen in all samples (Fig. 2F).

Infections were also performed *in vitro* in mouse peritoneal macrophages for transmission electron microscopy (infected host cells) and in the human monocytic cell line THP1 for scanning electron microscopy (amastigotes extracted from host cells). Transmission electron microscopy analysis of infected cells (Fig. 2G) shows that intracellular parasites from all three lines were tightly enclosed within the host cell and had an ovoid body shape characteristic of amastigotes. In amastigotes from the *BBS1* null line, there was no evidence of the vacuole-like structures seen around the flagellar pocket in the promastigote stage. Scanning electron microscopy imaging was also performed on parasites extracted from infected host cells (Fig. 2H). Characteristic amastigote morphology was observed in the three parasite lines, including the presence of a tiny flagellum emerging from the flagellar pocket.

Cumulatively, these data indicate that while BBS1 expression affects *L. major* infectivity of macrophages, it is not essential for attachment, early infection or differentiation from the metacyclic promastigote to the amastigote stage inside cultured macrophages.

### BBS1 is required for persistence in a mouse model of infection

**Figure 3:**
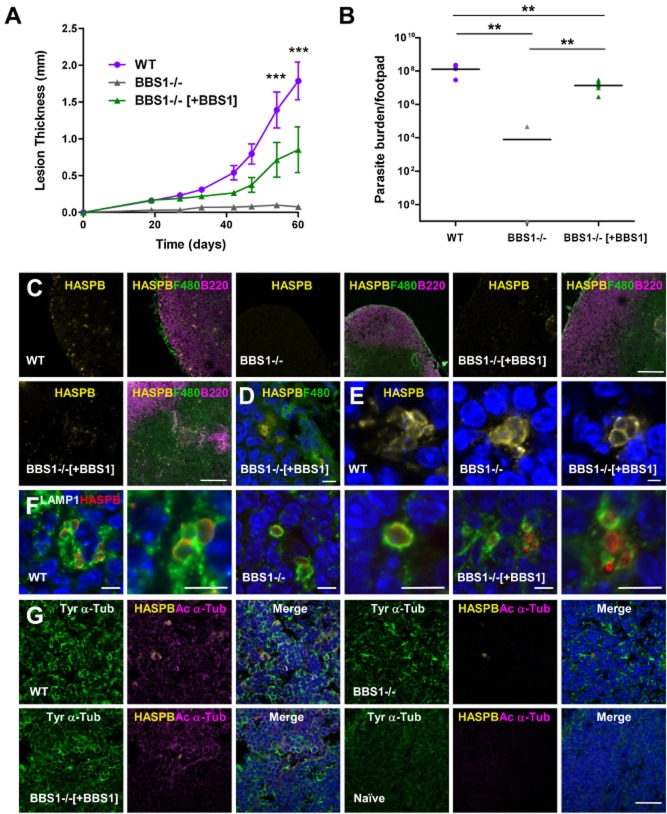
Effect of *BBS1* gene deletion on *L. major* host infectivity.A. BALB/c mice were infected with *L. major* wild-type (WT), *BBS1* null (BBS1−/−) and complemented (BBS1−/−[+BBS1]) lines by subcutaneous injection of 5 × 10^6^ metacyclic promastigotes into the right hind footpad. Developing lesions were monitored over 60 days. Mean lesion thickness is shown (*n* = 5) ± SD. Data presented here represent one of two independent experiments.B. Parasite burden was measured in three footpads from each group of infected mice as in (A), by a limiting dilution assay following termination. Mean parasite burden per footpad is shown, combining data from two independent experiments (*n* = 6) ± SD. By this method, no parasites were detected in five of six mice infected with the *L. major* *BBS1* null line.C. Immunofluorescence of lymph nodes draining the site of infection in BALB/c mice, 60 days post-infection with *L. major* parasite lines as above (two areas of the lymph node are shown for BBS1 complemented line). Tissue sections were probed with antibodies against *L. major* HASPB (yellow), the macrophage marker F4/80 (green) and B-cell marker B220 (pink). Bar, 200 μm.D. Lymph node section from a mouse infected with BBS1 complemented line for 60 days, probed with anti-HASPB (yellow) and F4/80 (green) and co-stained with DAPI (blue). Bar, 20 μm.E. Infected mouse lymph node sections probed with anti-HASPB (yellow) and co-stained with DAPI (blue). Bar, 2.5 μm.F. Infected mouse lymph node sections probed with anti-HASPB (red) and anti-LAMP1 (green) and co-stained with DAPI (blue). Bar, 5 μm.G. Infected and naïve mouse lymph node sections probed with anti-HASPB (yellow), anti-acetylated α-tubulin (pink) and anti-tyrosinated α-tubulin (green) and co-stained with DAPI (blue). Bar, 20 μm.

As *L. major* parasites lose virulence over time in culture, BBS1 mutant lines were passaged *in vivo* prior to the infection studies presented in Figs 2 and 3. This process requires administration of metacyclic promastigotes by subcutaneous injection into the right hind footpad of BALB/c mice. This mouse strain is susceptible to *L. major* infection and a non-healing cutaneous lesion develops over a period of weeks following injection of parasites. Amastigotes can be isolated either from the site of infection or from the lymph node draining this site, and then differentiated back to procyclic promastigotes in culture.

On first passage, very few *BBS1* null parasites were recovered from the draining lymph node at 4 weeks post-infection compared with wild-type and *BBS1* complemented lines (> 1000-fold difference, data not shown). To quantify this observation, experiments were performed using second passage metacyclic promastigotes to infect groups of five BALB/c mice, measuring footpad size weekly as an indicator of infection, followed by analysis of infecting parasite number. Lesion development was more rapid and extensive in mice infected with wild-type *L. major* parasites compared with the BBS1 mutant lines, with a highly significant difference in lesion size by 48 days onwards (Fig. 3A). Mice infected with the *BBS1* complemented line developed lesions by the later time points of the experiment but no footpad swelling was observed in animals infected with *BBS1* null parasites by 60 days post-infection when the experiment was terminated (Fig. 3A).

The parasite burden in the footpads of three mice from each group was determined by a limiting dilution assay. The combined results from two independent experiments are shown in Fig. 3B. The mean parasite burden per footpad for wild-type *L. major* was 1.27 × 10^7^ compared with 1.33 × 10^6^ per footpad for mice infected with the *BBS1* complemented line (*P* value < 0.01). In contrast, no parasites were detected in five out of six mice infected with the *L. major BBS1* null line. A persistent infection with the *BBS1* null line was found in only one mouse, in which the footpad parasite burden was 4.7 × 10^4^ (Fig. 3B). Therefore the absence of BBS1 has a severe detrimental effect on the ability of *L. major* parasites to infect and persist in a mouse model, while complementation with TdTomato-tagged BBS1 partially restores virulence.

This inability to persist in the host could be due to defects in amastigote proliferation, signalling mechanisms or nutrient metabolism, reduced ability of amastigotes to escape the macrophage and infect other host cells or weakened defence against the host immune system. As the BBSome is associated with protein trafficking in other organisms, it is possible that loss of BBS1 function has detrimental effects on multiple pathways. *Leishmania* parasites have evolved to rapidly respond to changes in environmental conditions, such as temperature, pH and nutrient availability, during progression through the life cycle. Procyclic promastigotes live in a carbohydrate-rich environment in the sand fly midgut and fulfil their energy requirements by uptake of hexose via transporters at the plasma membrane and flagellum (Jacobson *et al*., 2001; Burchmore *et al*., 2003). A reduction in hexose availability has been proposed to act as one of several triggers proposed to induce differentiation to the metacyclic stage as the parasite progresses to the salivary gland, prior to transmission to the host. Once inside the macrophage parasitophorous vacuole, the parasite encounters a hostile nutrient-poor environment with lower pH and higher temperature and must undergo a series of morphological and physiological changes in order to maintain viability. Differentiation to the amastigote form is linked to specific protein phosphorylation events, including the action of stress-induced PERK eIF2α kinase (*Leishmania infantum*) (Chow *et al*., 2011) and a MAP kinase kinase, PK4 (*Leishmania mexicana*) (Kuhn and Wiese, 2005). The surface glycoconjugate LPG is absent or very low abundance on the surface of amastigotes (Glaser *et al*., 1991; Bahr *et al*., 1993; Moody *et al*., 1993) and, correlating with this, is an important virulence factor in metacyclic promastigotes but not in amastigotes (Spath *et al*., 2000). In contrast, glycosylinositolphospholipids (GIPLs) are abundant on the surface of both promastigotes and amastigotes but are not required for amastigote infectivity (Zufferey *et al*., 2003). The most abundant protein on the parasite surface is the GPI-anchored protease gp63 which inactivates the p38 MAP kinase and mTORC1 signalling pathways during early infection and may also subvert host response from within the parasitophorous vacuole (Halle *et al*., 2009; Jaramillo *et al*., 2011).

Immunoblotting was performed using serum samples from infected mice to probe total lysate from *L. major* wild-type metacyclic promastigotes (Supplementary Fig. S6A). There was no evidence that the *BBS1* null had failed to survive due to high serum IgG in the host. A broad variation in antibody responses was observed across the samples analysed but these were overall stronger and had a wider reactivity pattern in the mice infected with wild-type *L. major* than the BBS1 mutant lines, correlating with a higher parasite burden. All mice responded to a parasite protein of approximately 50 kDa, indicating that an early infection occurred in all cases enabling antigenic exposure. Serum samples predominantly recognizing this 50 kDa band were used to probe wild-type *L. major* promastigotes (Supplementary Fig. S6B). These sera strongly labelled the cell surface and flagellum, suggesting that the 50 kDa antigen is one or more isoforms of tubulin.

The lymph nodes draining the site of infection in infected mice were subjected to immunofluorescence analysis with antibodies against *L. major* HASPB, the macrophage marker F4/80 and B-cell marker B220 to delineate their cellular architecture. Lymph node hypertrophy was apparent in mice infected with *L. major* wild-type and the *BBS1* complemented line but not in those infected with the *BBS1* null line (data not shown). In mice infected with wild-type *L. major*, parasites were visible throughout the lymph node and were particularly dense in the regions corresponding to the subcapsular macrophages (Fig. 3C). Fewer *BBS1* complemented parasites were detected in the lymph node compared with wild-type, mainly localizing to F4/80-positive cells in the inner cortex (Fig. 3C and D). No parasites were observed in the lymph nodes of mice infected with the *L. major BBS1* null line with the exception of the single mouse showing a positive result in the limiting dilution assay described above, in which < 10 parasites were detected per 10 μm section of lymph node (Fig. 3C). High-resolution fluorescence images show that intracellular parasites from all three parasites lines had similar gross morphology, with HASPB correctly trafficked to the plasma membrane in all cases (Fig. 3E). HASPB could also be observed in small foci around amastigotes from all lines, providing further evidence that this acylated protein is released into the host environment (Maclean *et al*., 2012). The lysosomal LAMP1 was recruited to the parasitophorous vacuole membrane in lymph nodes infected with the three parasite lines, with no clear differences between the samples (Fig. 3F). Immunofluorescence was also used to analyse post-translational modifications of host tubulin in the infected lymph nodes (Fig. 3G). While tyrosinated α-tubulin was found at a similar level in all infected samples, the abundance of acetylated α-tubulin was increased in lymph nodes infected with *L. major* wild-type and *BBS1* complemented lines. This increase was seen throughout the lymph node section and not restricted to areas of infected cells, suggesting that this is a non-specific effect correlating with inflammation rather than a specific response induced by intracellular parasites. Samples infected with *L. major BBS1* null parasites had a low level of this type of modified tubulin, similar to that seen in lymph nodes from naïve mice (Fig. 3G), correlating with a lack of inflammation and low parasite burden in *BBS1* null infected samples, as compared with control infections.

### Does BBS1 affect the function of the amastigote flagellum?

During differentiation from promastigote to amastigote, the long motile flagellum is considerably shortened but whether this is due to resorption or shedding has not been established. Amastigotes were historically described as aflagellated but electron microscopy revealed that this stage of the parasite has a very short flagellum with a 9 + 0 microtubule pair configuration resembling that of primary cilia, rather than the 9 + 2 pattern seen in the motile flagella of promastigotes (Alexander, 1978; Gluenz *et al*., 2010). If this rudimentary flagellum formed a tight junction with the parasitophorous vacuole membrane, this could be a potential route for exchange of material by membrane fusion. Alternatively, the amastigote flagellum could act as a secretory organelle as observed in *Chlamydomonas* (Baldari and Rosenbaum, 2010). Further work will be necessary to address these questions.

Our data presented here suggest that *L. major* BBS1 has an amastigote-specific role in host virulence. We currently have no direct evidence that the BBSome in *L. major* has a role in flagellar transport, although observations in other species would suggest that this is likely to be the case. If true, this would suggest that the amastigote flagellum plays a key role in survival of the intracellular parasite and that a BBSome-dependent pathway exists for transport of critical molecules required for parasite viability. Confirmation of this mechanism and identification of its cargo molecules may provide valuable insights into the intracellular survival of this important human pathogen.

## Experimental procedures

### DNA constructs

Parasite genomic sequences were obtained from TriTrypDB (Aslett *et al*., 2010). Detailed methods for generation of DNA constructs and qRT-PCR are provided in Supplementary Experimental Procedures. Primer sequences are shown in Supplementary Table S1.

### Parasite culture and transfection

*Leishmania major* (MHOM/IL/81/Friedlin) promastigotes were maintained *in vitro* at 26°C as described (Flinn *et al*., 1994). Parasites were routinely seeded at 5 × 10^4^ ml^−1^ and incubated at 26°C for 2 days to produce log-phase procyclic promastigotes, or for 7 days to produce metacyclic promastigotes. To generate knockout lines, linear targeting regions were produced by digesting pBBS1-KO-HYG and pBBS1-KO-PAC (see Supplementary Experimental Procedures) with HindIII/BglII. Following purification, the fragments were used sequentially to transfect mid-log phase *L. major* promastigotes by nucleofection, as described (Brannigan *et al*., 2010). Transfected parasites were selected with 32 μg ml^−1^ hygromycin (Life Technologies) and 20 μg ml^−1^ puromycin (Sigma) as appropriate. For complementation of null lines, transfection was performed as above using linearized pSSU-BBS1-Tom (digested with PacI/PmeI) or circular pTEX-BBS1-V5. Cells were selected with 40 μg ml^−1^ neomycin (Life Technologies). For expression of GFP-tagged IFT proteins, *L. major* wild-type and *BBS1* null lines were transfected with pSSU-IFT27-GFP (digested with PacI/PmeI) or pTEX-IFT52-GFP and selected with 40 μg ml^−1^ neomycin.

Southern hybridization to test for correct genomic integration was performed using methods described previously (Brannigan *et al*., 2010). Parasite growth was analysed using a Beckman Coulter counter. Transgenic *L. major* lines were passaged once through BALB/c mice (see below) before further analysis and all lines used in experiments described here were maintained in culture for < 10 passages.

### Microscopy

*Leishmania* parasites were fixed in 4% paraformaldehyde at RT for 15 min (or in 100% methanol at −20°C for 5 min for IFT172 staining), then indirect immunofluorescence assays were performed as described previously for *T. brucei* (Price *et al*., 2010b) with detection using Alexa Fluor 488- and 594-conjugated secondary antibodies (Life Technologies). Primary antibodies used were: mouse monoclonal antibody TAT1 against *T. brucei* α-tubulin (1:200, a gift from Keith Gull, Sir William Dunn School of Pathology, University of Oxford, UK), mouse monoclonal antibody against *T. brucei* IFT172 (Absalon *et al*., 2008) (1:200, a gift from Philippe Bastin, Pasteur Institute, Paris, France), mouse monoclonal antibody clone 6-11B-1 against acetylated α-tubulin (1:300, Sigma), rabbit anti-RFP/dsRed (1:200, Abcam), mouse monoclonal anti-V5 (1:200, Life Technologies) and rabbit anti-HASPB_336_ (Flinn *et al*., 1994) (1:300). Production of a rabbit polyclonal antibody against *L. major* paraflagellar rod protein 1 (PFR1) is described in Supplementary Experimental Procedures. Live imaging of TdTomato-BBS1, IFT52-GFP and IFT27-GFP proteins was performed by immobilization in Cygel as described previously (Price *et al*., 2010a).

In order to distinguish morphological forms, *L. major* promastigotes were grown in culture for 7 days before staining with TAT1 and LmPFR1. Cell body length, width and flagellum length were measured from acquired images using LSM 510 v3.2 software (Zeiss). Morphological forms were assigned as previously (Walters, 1993; Cihakova and Volf, 1997) by the following criteria: (i) short promastigotes: body length < 14 μm and flagellum length < 2 times body length, (ii) elongated nectonomads: body length ≥ 14 μm, (iii) metacyclic promastigotes: body length < 14 μm and flagellum length ≥ 2 times body length, and (iv) round forms: body width > 4 μm and body length ≤ 7.5 μm. Staining with FM4-64FX (Life Technologies) was performed as described previously (Price *et al*., 2010a). Transmission electron microscopy was performed as described previously (Price *et al*., 2003). Scanning electron microscopy was performed as described for *T. brucei* (Price *et al*., 2010b).

### Osmotaxis assay

The ability of parasites to respond to a sucrose gradient was assayed as described previously (Leslie *et al*., 2002). Briefly, promastigotes were harvested at day 3 post-inoculation and washed in WIS buffer (30 mM sodium β-glycerophosphate, 87 mM NaCl, 27 mM KCl, 2 mM MgCl_2_, pH 7.1, 0.004% BSA). Cells were resuspended in WIS buffer at a concentration of 2.5 × 10^7^ ml^−1^. Glass capillary tubes (75 mm length, 0.8 mm inner/1 mm outer diameter) were prepared, containing 100 mM sucrose in 1% agarose, leaving 1 cm free at the end of each tube which was then filled with WIS buffer. Prepared capillary tubes were incubated in a Petri dish with washed parasites at 25°C for 30 min. The number of parasites per capillary tube was counted using a haemocytometer. Six capillary tubes were prepared for each sample and the attraction coefficient was calculated as the number of parasites migrating to capillary tubes containing sucrose divided by the number migrating into control capillary tubes containing agarose alone.

### Proteomic analysis of flagella extracts

Detailed methods for extraction and analysis of *L. major* flagella are provided in Supplementary Experimental Procedures and identified proteins are listed in Supplementary Table S2.

### Macrophage infections

Murine BALB/c peritoneal macrophages were obtained by peritoneal lavage with RPMI 1640 medium (Sigma) after 24 h induction with 2% starch solution (Sigma). Macrophages were seeded with RPMI 1640 medium/10% FCS at 3 × 10^5^ cells per well onto 24-well plates containing acetone treated glass coverslips and incubated overnight at 37°C with 5% CO_2_. *L. major* metacyclic promastigotes were added to macrophages at a ratio of 10:1, plates were centrifuged at 1700 *g* for 5 min at RT and incubated at 34°C with 5% CO_2_ for 2 h, before washing in RPMI 1640 medium. Cells were then incubated at 34°C with 5% CO_2_ for up to 96 h before fixation with 100% methanol for 5 min at RT (w/v). Indirect immunofluorescence analysis was performed as described (Maclean *et al*., 2012). For transmission electron microscopy, infections were performed in 25 cm^2^ flasks using mouse peritoneal macrophages. Cells were incubated for 72 h at 34°C with 5% CO_2_, and then harvested by scraping. Transmission electron microscopy was then performed as described previously (Price *et al*., 2003). For scanning electron microscopy of amastigotes, infections were performed in 25 cm^2^ flasks using the human monocytic cell line THP1. Cells were incubated for 72 h at 34°C with 5% CO_2_, then amastigotes harvested as described (Jain *et al*., 2012). Scanning electron microscopy was then performed as described for *T. brucei* (Price *et al*., 2010b).

### Mouse infections

Animal experiments were approved by the University of York Animal Procedures and Ethics Committee and performed under UK Home Office licence (‘Immunity and Immunopathology of Leishmaniasis’ Ref # PPL 60/3708). For routine *in vivo* passaging of parasites, BALB/c mice were infected by subcutaneous injection of 5 × 10^6^ *L. major* metacyclic promastigotes into the right hind footpad. Infections were terminated after 4 weeks and *ex vivo* draining lymph nodes were incubated at 26°C in M199 medium until the emergence of *L. major* promastigotes. For analysis of parasite virulence, groups of five mice were infected as above and the widths of the right infected and left uninfected footpads were measured weekly using direct reading Vernier callipers. Experiments were terminated once cutaneous lesions were evident (∼ 2 mm thickness) in any of the groups of mice. Parasite burden in footpads was measured by a limiting dilution assay as described (Titus *et al*., 1985; Lima *et al*., 1997).

For immunoblotting, wild-type *L. major* promastigotes (day 7) were lysed in 1× Laemmli buffer, separated by SDS-PAGE and transferred to nitrocellulose. Immunoblots were probed with infected mouse serum samples (diluted 1:200), followed by goat anti-mouse HRP (1:25 000, Sigma) and developed using ECL Prime (GE Healthcare Life Sciences). For indirect immunofluorescence, draining lymph node sections (10 μm thick) were fixed with 4% paraformaldehyde (w/v) and stained as described (Yurdakul *et al*., 2011) using Alexa Fluor 488-conjugated anti-mouse F4/80 (1:200, AbDSerotec), Alexa Fluor 647-conjugated anti-mouse B220 (1:200, AbDSerotec), mouse monoclonal 6-11B-1 against acetylated α-tubulin (1:300, Sigma), rat monoclonal YL1/2 against tyrosinated α-tubulin (1:200, Abcam), mouse monoclonal H4A3 against LAMP1 (1:500, Abcam) and rabbit anti-HASPB_336_ (Flinn *et al*., 1994) (1:300). Alexa Fluor 488-, 555-, 594- and 633-conjugated secondary antibodies were used for detection where appropriate (Life Technologies).

### Statistical analysis

Data shown represent one of three independent experiments unless otherwise stated. Statistical analysis was performed with GraphPad Prism 4 software, using either one-way anova (Tukey's multiple comparison test) or Student's *t*-test as appropriate, with *P* < 0.05 considered significant. Mean values are shown and error bars represent standard error unless otherwise stated.
