# Supplementary material for: The Leishmania major BBSome subunit BBS1 is essential for parasite virulence in the mammalian host
Source: Mol Microbiol. 2013 Sep 17;90(3):597–611. doi: 10.1111/mmi.12383 (PMC3916885; doi:10.1111/mmi.12383)
Supplement: Supplementary file 1 [file mmi0090-0597-sd1.zip › mmi_12383_sm_Experimental-Procedures.docx]

**Supplementary Experimental Procedures**

*DNA constructs*

All primer sequences are provided in Supplementary Table 1. For production of *BBS1* null *L. major* lines, a 602 bp region of *L. major* genomic DNA beginning 703 bp upstream of the *BBS1* ORF (LmjF.35.4180,TriTrypDB version 3.3) was amplified using primers BBS1-F1 and BBS1-R1, digested with *Hind*III/*Sal*I, then ligated into *Hind*III/*Sal*I digested pNMT-HYG ([Price *et al.*, 2003](#_ENREF_5)) to produce pBBS1-5’-HYG. A 728 bp region beginning 371 bp downstream of the *BBS1* ORF was then amplified from genomic DNA using primers BBS1-F2 and BBS1-R2, digested with *Bgl*II/*Xma*I and ligated into *Bgl*II/*Xma*I digested pBBS1-5’-HYG to produce the knockout construct pBBS1-KO-HYG. To generate plasmid pBBS1-PAC, the *PAC* gene was released from plasmid plmcpb-PAC ([Mottram *et al.*, 1996](#_ENREF_3)) as previously described ([Price et al., 2003](#_ENREF_5)) and used to replace the *HYG* gene in pBBS1-HYG, following *Spe*I/*Bam*HI digestion, to produce the construct pBBS1-KO-PAC. For complementation, the BBS1 ORF (1779 bp) was amplified from *L. major* genomic DNA using the primers BBS1-F3 and BBS1-R3. The fragment was digested with *Xho*I/*Not*I and ligated into vector pSSU-Neo-tdTomato to produce the construct pSSU-BBS1-Tom, which integrates at the rDNA locus in the *L. major* genome and encodes the BBS1 protein with an N-terminal tdTomato tag. For expression of BBS1 with a C-terminal V5 epitope tag, the BBS1 ORF (1779 bp) was amplified from genomic DNA using primers BBS1-F4 and BBS1-R4 and ligated into the plasmid vector pET101/D-TOPO (Life Technologies) to produce the construct pET101-BBS1-V5. The BBS1 ORF fused to a V5 epitope tag was then amplified from this construct using primers BBS1-F5 and BBS1-R5. The fragment was digested with *Eco*RI/*Hind*III and cloned into *Eco*RI/*Hind*III cut expression vector pTEX to produce the construct pTEX-BBS1-V5.

For expression of GFP-tagged IFT52, a 2019 bp region corresponding to the ORF of *L. major* IFT52 (LmjF.19.0320) was amplified from genomic DNA using primers IFT52-F1 and IFT52-R1 and ligated into pcDNA3.1/CT-GFP-TOPO (Life Technologies). Tagged-IFT52 was then amplified from this construct using primers IFTGFP-F1 and IFTGFP-R1. A 2.7 kb fragment was ligated into *Eco*RV-cut pTEX to produce the construct pTEX-IFT52-GFP for episomal transfection. For expression of GFP-tagged IFT27, a 600 bp region corresponding to the ORF of *L. major* IFT27 (LmjF.29.0090) was amplified from genomic DNA using primers IFT27-F1 and IFT27-R1 and ligated into pcDNA3.1/CT-GFP-TOPO (Life Technologies). Tagged-IFT27 was then amplified from this construct using primers IFTGFP-F1 and IFTGFP-R2. A 1.3 kb fragment was digested with *Bgl*II/*Kpn*I and ligated into *Bam*HI/*Kpn*I cut vector pSSU-Neo to produce the construct pSSU-IFT27-GFP for genomic integration at the rDNA locus.

*Quantitative RT-PCR*

Absolute quantitation by qPCR was used to determine the levels of *BBS1*-specific transcript in *L. major* life cycle stages and transgenic lines, relative to a constitutively expressed control, myristoyl-CoA:protein N-myristoyltransferase (NMT). Total RNA was extracted from parasites using Trizol reagent (Life Technologies) and traces of genomic DNA were removed by treatment with DNase I. Reverse transcription was performed using Omniscript RT (Qiagen) and Oligo-dT (Promega). The program Primer Express (Applied Biosystems) was used to design the following primers: BBS1-qPCR-F1, BBS1-qPCR-R1, NMT-qPCR-F1 and NMT-qPCR-R1 (Supplementary Table 1). Quantitative PCR was carried out using Power SYBR Green PCR Mastermix (Applied Biosystems) on an ABI 7300 Sequence Detection System (Applied Biosystems) and results detected with Sequence Detection Software v1.2.3 (Applied Biosystems).

*Production of Rabbit Polyclonal Antibody against L. major PFR1*

The full open reading frame of *L. major* paraflagellar rod protein 1D (PFR1D, LmjF.29.1750) was amplified from *L. major* genomic DNA, cloned into plasmid vector pET-YSBLIC3C (pET28a modified for ligation-independent cloning) ([Alzari *et al.*, 2006](#_ENREF_1)) and transformed into *E.coli* Rosetta 2. Expression of the C-terminal His-tagged protein was achieved in autoinduction medium with overnight growth at 30°C. Cells were resuspended in lysis buffer (300 mM NaCl, 20 mM sodium phosphate pH 7.5, 20 mM imidazole, protease inhibitors and DNAse I) and passed through a continuous flow French Press. The crude lysate was clarified by centrifugation at 50,000 *g* for 40 minutes at 4°C followed by filtration through a 0.8 μm membrane. Protein purification was performed using Ni2+-affinity/size exclusion multi-dimensional liquid chromatography on an ÄKTA Express (GE Healthcare). Final fractions were analysed by SDS-PAGE. Polyclonal antibodies were produced from two rabbits using the Eurogentech 87 day Classic protocol. Antibodies were purified using a 1ml NHS-activated HP column (GE) coupled with 1 mg recombinant LmPFR1 protein. Rabbit serum was loaded onto the column in binding buffer (20 mM sodium phosphate pH7, 150 mM NaCl), washed with the same buffer and then eluted with low pH buffer (0.1 M glycine pH2.7, 0.5 M NaCl). Fractions were collected into tubes containing 1M Tris-HCl pH9 for neutralisation. Fractions were analysed by immunoblotting and immunofluorescence. Following immunoblotting of total *L. major* promastigote lysate, purified antibody (1:2000 dilution) recognised a single protein band of 69 kDa corresponding to the PFR protein.

*Proteomic Analysis of Flagella Extracts*

*2D gel electrophoresis*

Flagella were isolated from *L. major* promastigotes by detergent/NaCl extraction as described previously for *T. brucei* ([Broadhead *et al.*, 2006](#_ENREF_2), [Price *et al.*, 2012](#_ENREF_4)). Pellets were solubilised in 2D extraction buffer (6M Urea, 2M Thiourea and 2% CHAPS/2% SB3-10) for 1 hour at RT, then centrifuged at 20,000 *g* for 30 mins. For each gel, 270 µg of solubilised protein was diluted to a volume of 460 µl in 2D sample buffer (extraction buffer with the addition of 1% IPG buffer (BioRad), 1% DeStreak reagent (GE) and a trace of bromophenol blue). Each sample was applied to separate wells of a rehydration tray and a 24 cm pH 3-10 IPG strip (GE) was placed gel side down in each well. Each strip was covered with DryStrip cover fluid (GE) prior to rehydration overnight at RT. IEF was then carried out using an IPGphor platform (GE) with the running conditions: IEF at 20ºC and 50μA per strip, 500V step-n-hold for 1 hour, 1000V gradient for 2 hours, 8000V gradient for 6 hours, 8000V step-n-hold for 60,000Vhrs. Following IEF, strips were incubated in equilibration buffer (6M urea, 34.5% (v/v) glycerol, 0.05M Tris, 2% (w/v) SDS, 1% (w/v) DTT) for 15 mins, then in equilibration buffer containing 4% iodoacetamide for 15 mins.

Equilibrated strips were applied to the top of 15% acrylamide gels, along with an electrode wick containing 20 μl Precision Plus prestained standards (BioRad). Gels were loaded into an Ettan Dalttwelve electrophoresis unit with 1x TGS buffer in the lower reservoir and 2x TGS in the upper reservoir. Electrophoresis was performed at 16 mA per gel for 30 mins and then 40 mA per gel for 6-7 hours. Gels were incubated in fixing solution (40% methanol, 10% acetic acid) for 1 hour, then overnight at RT in Sypro Ruby solution (Molecular Probes). Gels were incubated for 1 hour in destain solution (10% methanol/ 7% acetic acid), then briefly in water. Imaging was performed on a Molecular Imager FX (BioRad). Gels were produced in triplicate and the resulting images analysed using ProGenesis Samespots software v3.2 (Nonlinear Dynamics) in order to identify expression differences between sample sets relative to a selected reference gel.

*Protein Identification by MS/MS*

Protein spots were excised from Coomassie stained gels, destained and digested with trypsin following standard protocols. The resulting peptide solutions were de-salted using µ-C18 Zip Tips (Millipore) following manufacturers’ guidelines. MS/MS analysis was performed on a Bruker Ultraflex. MS spectra were acquired in the mass range m/z 800-4000. The 10 most intense peaks present on the MS spectra for each sample were then submitted for MS/MS analysis. The mass spectral data were submitted for database searching against the NCBInr database using a locally running copy of the Mascot software (Matrix Science) through a Biotools (Bruker Daltonics) interface.

**References**

Alzari, P. M., H. Berglund, N. S. Berrow, E. Blagova, D. Busso, C. Cambillau, V. Campanacci, E. Christodoulou, S. Eiler, M. J. Fogg, G. Folkers, A. Geerlof, D. Hart, A. Haouz, M. D. Herman, S. Macieira, P. Nordlund, A. Perrakis, S. Quevillon-Cheruel, F. Tarandeau, H. van Tilbeurgh, T. Unger, M. P. Luna-Vargas, M. Velarde, M. Willmanns & R. J. Owens, (2006) Implementation of semi-automated cloning and prokaryotic expression screening: the impact of SPINE. *Acta Crystallogr D Biol Crystallogr* 62: 1103-1113.

Broadhead, R., H. R. Dawe, H. Farr, S. Griffiths, S. R. Hart, N. Portman, M. K. Shaw, M. L. Ginger, S. J. Gaskell, P. G. McKean & K. Gull, (2006) Flagellar motility is required for the viability of the bloodstream trypanosome. *Nature* 440: 224-227.

Mottram, J. C., A. E. Souza, J. E. Hutchison, R. Carter, M. J. Frame & G. H. Coombs, (1996) Evidence from disruption of the lmcpb gene array of Leishmania mexicana that cysteine proteinases are virulence factors. *Proc Natl Acad Sci U S A* 93: 6008-6013.

Price, H. P., M. R. Hodgkinson, R. S. Curwen, L. M. MacLean, J. A. Brannigan, M. Carrington, B. A. Smith, D. A. Ashford, M. Stark & D. F. Smith, (2012) The orthologue of Sjogren's syndrome nuclear autoantigen 1 (SSNA1) in Trypanosoma brucei is an immunogenic self-assembling molecule. *PLoS One* 7: e31842.

Price, H. P., M. R. Menon, C. Panethymitaki, D. Goulding, P. G. McKean & D. F. Smith, (2003) Myristoyl-CoA:protein N-myristoyltransferase, an essential enzyme and potential drug target in kinetoplastid parasites. *J Biol Chem* 278: 7206-7214.
