# Supplementary material for: The Leishmania major BBSome subunit BBS1 is essential for parasite virulence in the mammalian host
Source: Mol Microbiol. 2013 Sep 17;90(3):597–611. doi: 10.1111/mmi.12383 (PMC3916885; doi:10.1111/mmi.12383)
Supplement: Supplementary file 1 [file mmi0090-0597-sd1.zip › mmi_12383_sm_FigureS1-6.pdf]

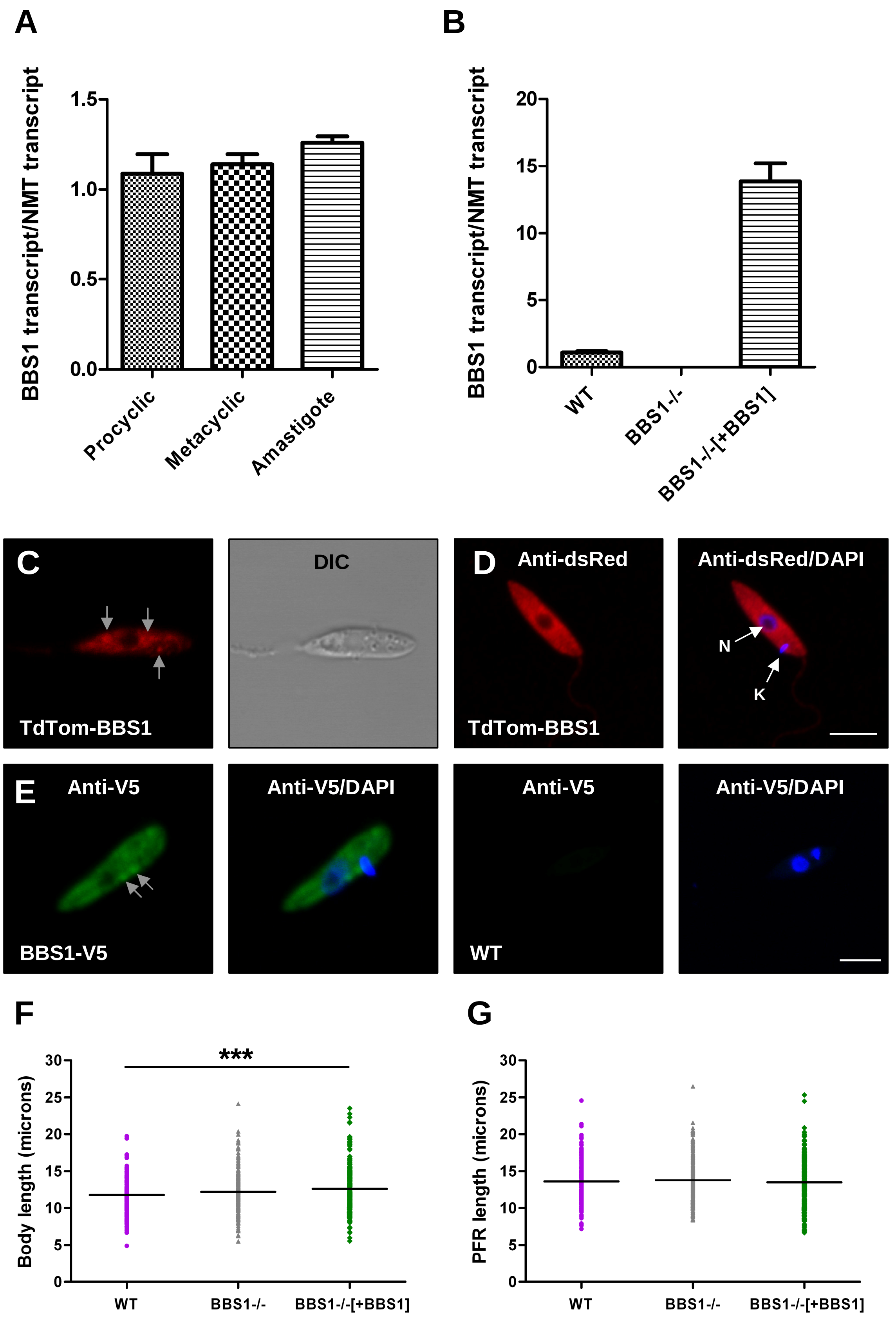

Supplementary Figure 1

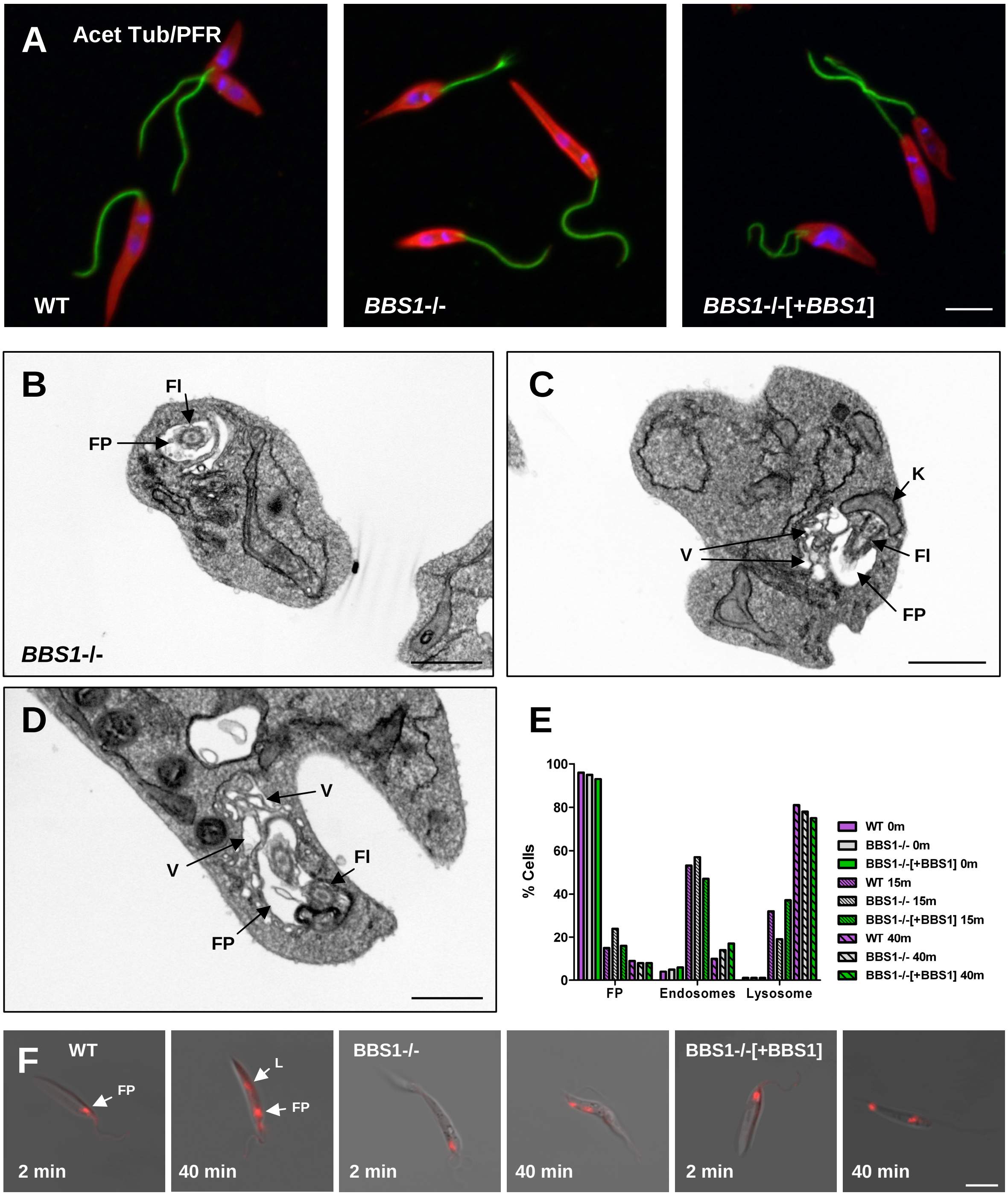

Supplementary Figure 2

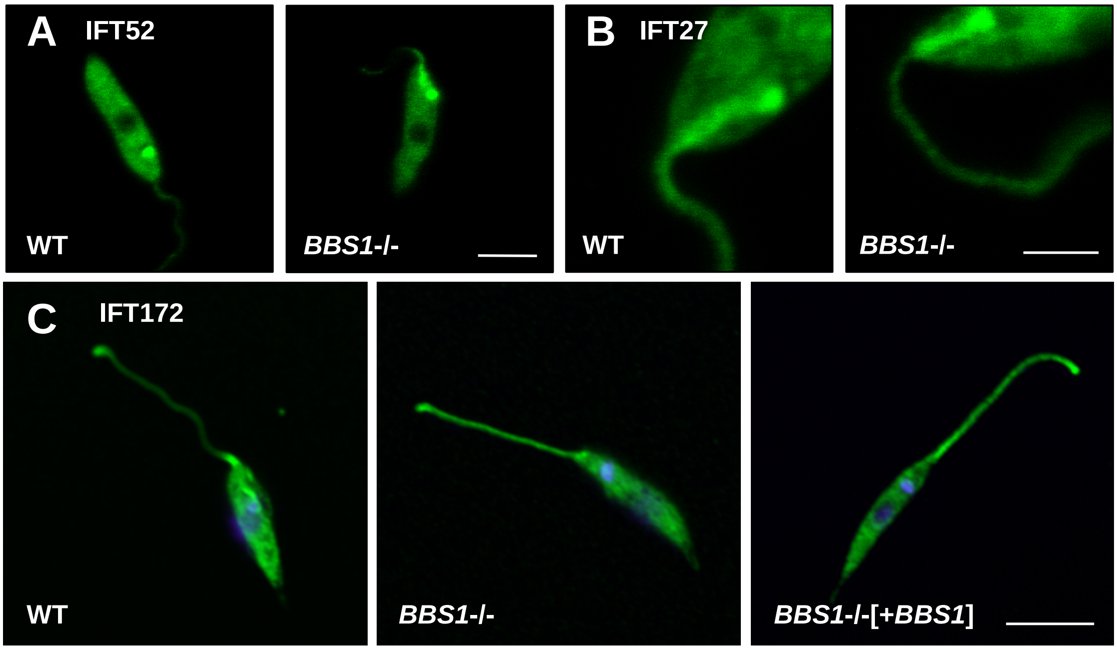

Supplementary Figure 3

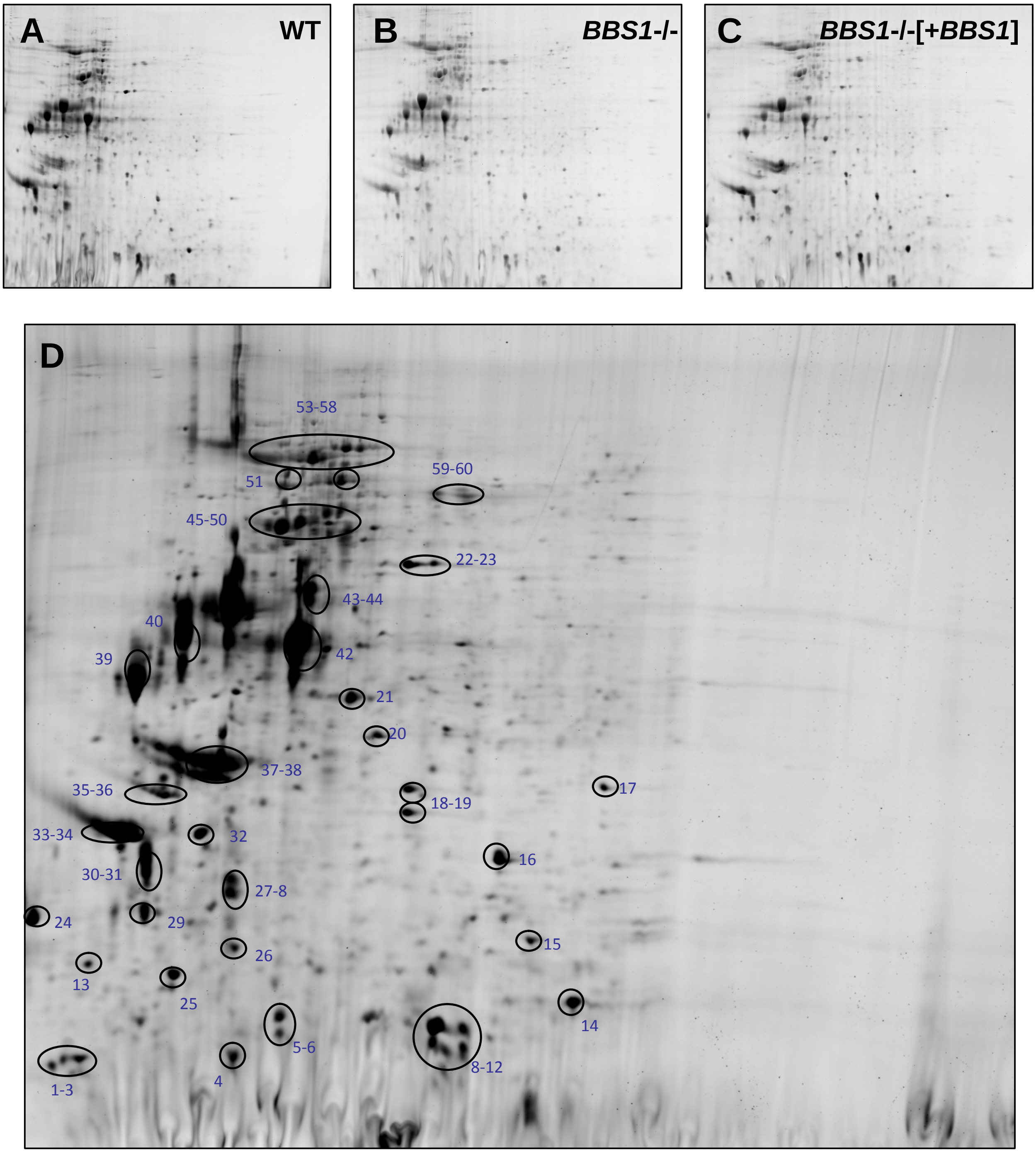

Supplementary Figure 4

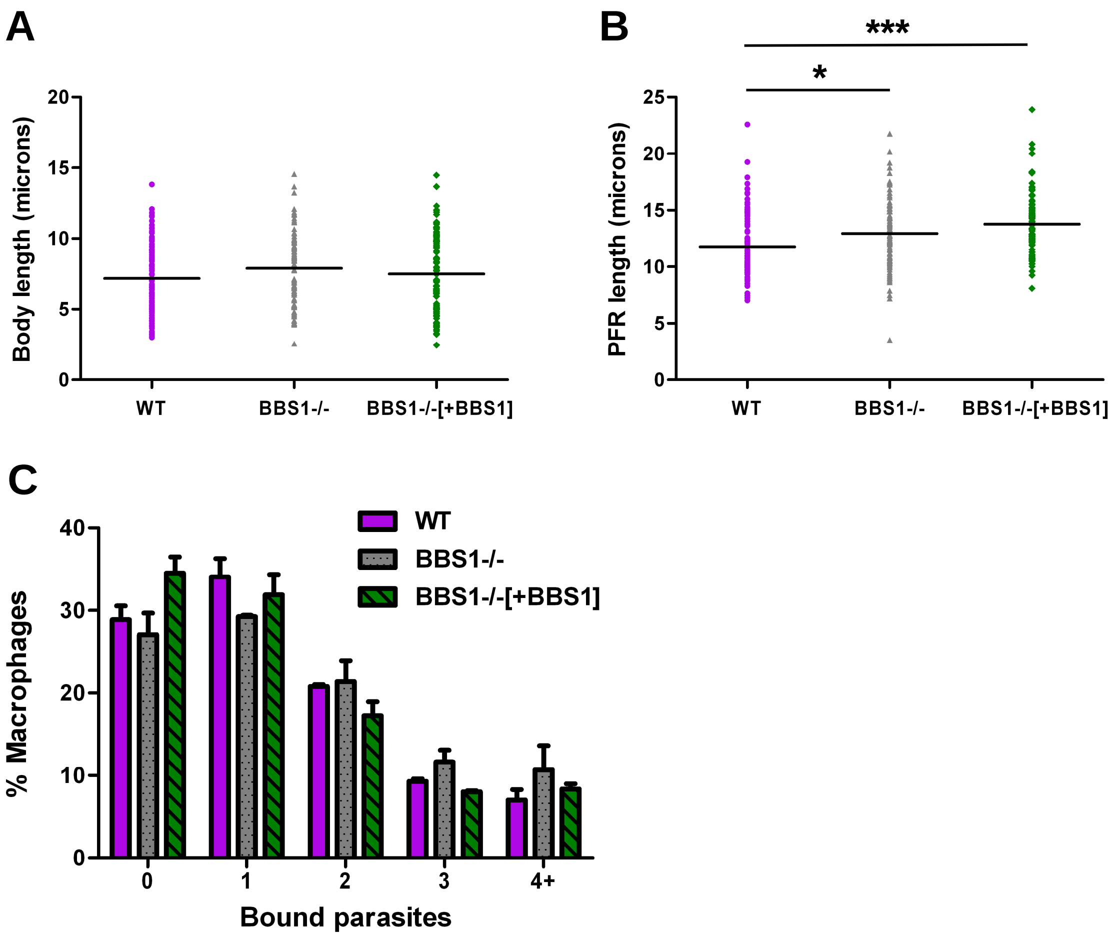

Supplementary Figure 5

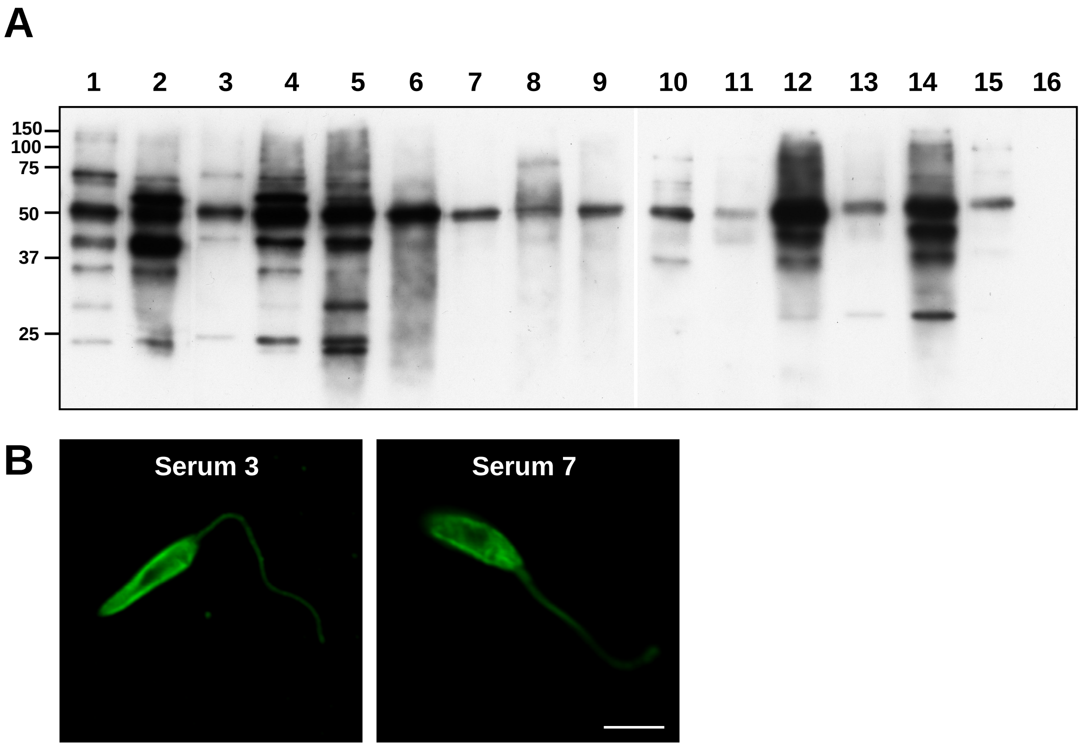

Supplementary Figure 6
