# Supplementary material for: The Leishmania major BBSome subunit BBS1 is essential for parasite virulence in the mammalian host
Source: Mol Microbiol. 2013 Sep 17;90(3):597–611. doi: 10.1111/mmi.12383 (PMC3916885; doi:10.1111/mmi.12383)
Supplement: Supplementary file 1 [file mmi0090-0597-sd1.zip › mmi_12383_sm_TableS1-2.pdf]

Primer	Sequence
BBS1-F1	5′-GTGCTCAGTTTCCGTAAAGCTTGTCTAAGGCAT-3′
BBS1-R1	5′-CAACTCGCCCACGTCGACAGGTGCG-3′
BBS1-F2	5′-GACGGAGGACCCGGGAGTAAGACACTGTGAAA-3′
BBS1-R2	5′-AGAGAGTTTCATAGAGGTAGCAGATCTCCTCAGC-3′
BBS1-F3	5′-GAGCCTCGAGCATGTCTGCACCCAAGGAGG-3′
BBS1-R3	5′-CCGGCGGCCGCTCACTTCTGCCATTGCTTC-3′
BBS1-F4	5′-CACCATGTCTGCACCCAAG-3′
BBS1-R4	5′-GAGGTGGAAGCAATGGCAGAAGTG-3′
BBS1-F5	5′-ACTTTAAGAAGGAATTCAGGAGCCCTTCAC-3′
BBS1-R5	5′-CTTCCTTTCAAGCTTTGTTAGCAGCCGGAT-3′
BBS1-qPCR-F1	5′-CAACCTGCAAAACGCTGTGA-3′
BBS1-qPCR-R1	5′-TGTTGTACCGCGCAATGG-3′
NMT-qPCR-F1	5′-GCCAAAGACGGTAGCCGATA-3′
NMT-qPCR-R1	5′-GGCGTCCACCACTCAAATGT-3′
IFT52-F1	5′-ATGACGGAGGTGACTTCACCATACCGTG-3′
IFT52-R1	5′-CCAGCTCTTCGAGCGTATATAGGAAGTTGC-3′
IFT27-F1	5′-ATGGTGCTCCTGCGCCTCCGCG-3′
IFT27-R1	5′-CCTTACTTGCGCTGAGCTGTGACAACTG-3′
IFTGFP-F1	5′-GAGCTCAGATCTACTAGTCCAGTGTGG-3′
IFTGFP-R1	5′-CAGTCGAGGCAGATCTGCGGGTTTAATTC-3′
IFTGFP-R2	5′-CTAGAAGGTACCGTCGAGGCTGATCAG-3′

Supplementary Table 1

Spot No	Protein ID	MOWSE score	Peptide Count	Species	Mass (kDa)	Accession number
**1**	beta tubulin	85	1	*L. major*	50.3	CAA63779
**26**	beta tubulin	89	1	*L. major*	50.3	CAA63779
**27**	beta tubulin	89	1	*L. major*	50.3	CAA63779
**28**	beta tubulin	202	4	*L. major*	50.3	CAA63779
**29**	beta tubulin	511	8	*L. major*	50.3	CAA63779
**30**	beta tubulin	177	3	*L. major*	50.3	CAA63779
**31**	beta tubulin	172	3	*L. major*	51.3	CAA63779
**34**	beta tubulin	109	2	*L. major*	50.3	CAA63779
**35**	beta tubulin	147	3	*L. major*	50.3	CAA63779
**36**	beta tubulin	70	2	*L. major*	50.3	CAA63779
**37**	beta tubulin	272	4	*L. major*	50.3	CAA63779
**38**	beta tubulin	230	4	*L. major*	50.3	CAA63779
**39**	beta tubulin	402	7	*L. major*	50.3	CAA63779
**40**	beta tubulin	243	4	*L major*	50.3	CAA63779
**43**	beta tubulin	236	3	*L major*	50.3	CAA63779
**58**	beta tubulin	238	3	*L major*	50.3	CAA63779
**4**	alpha tubulin	217	3	*L. donovani*	50.5	AAA58321
**9**	alpha tubulin	433	3	*L.donovani*	50.5	AAA58321
**10**	alpha tubulin	327	3	*L. donovani*	50.5	AAA58321
**12**	alpha tubulin	97	2	*L. donovani*	50.5	AAA58321
**27**	alpha tubulin	213	2	*L. donovani*	50.5	AAA58321
**28**	alpha tubulin	61	1	*L. donovani*	50.5	AAA58321
**42**	alpha tubulin	147	2	*L. donovani*	50.5	AAA58321
**43**	alpha tubulin	497	4	*L. donovani*	50.5	AAA58321
**44**	alpha tubulin	640	5	*L. donovani*	50.5	AAA58321
**54**	alpha tubulin	257	2	*L. donovani*	50.5	AAA58321
**59**	alpha tubulin	120	2	*L. donovani*	50.5	AAA58321
**60**	alpha tubulin	199	2	*L. donovani*	50.5	AAA58321
**2**	Hypothetical protein	174	1	*L. major*	11.8	XP_001686942
**5**	OMPDCaseOPRTase	47	1	*L. major*	50.0	XP_001682133
**6**	PFR 2C	85	1	*L. major*	69.8	XP_001682225
**7**	SMP1	136	2	*L. major*	15.5	2FE0_A
**8**	KMP11	126	2	*L. major*	11.2	XP_843327
**9**	KMP11	55	1	*L.major*	11.2	XP_843327
**11**	KMP11	119	2	*L. major*	11.2	XP_843327
**12**	KMP11	111	2	*L. major*	11.2	XP_843327
**13**	Centrin	231	4	*L. major*	16.4	XP_001683292
**14**	EF1alpha	141	2	*L. major*	44.1	XP_001682258
**15**	PFR1D	81	2	*L. major*	69.4	XP_847841
**21**	PFR1D	389	4	*L. major*	69.4	XP_847841
**32**	PFR1D	186	2	*L. major*	69.4	XP_847841
**45**	PFR1D	407	6	*L. major*	69.4	XP_847842
**46**	PFR1D	453	6	*L. major*	69.4	XP_847842
**47**	PFR1D	459	6	*L. major*	69.4	XP_847842
**48**	PFR1D	127	2	*L. major*	69.4	XP_847842
**53**	PFR1D	221	3	*L. major*	69.4	XP_847842
**16**	PFR 2C	265	3	*L. major*	69.8	XP_001682225
**17**	PFR 2C	259	4	*L. major*	69.8	XP_001682225
**49**	PFR2C	477	5	*L. major*	69.8	XP_001682225
**50**	PFR2C	408	7	*L. major*	69.8	XP_001682225
**52**	PFR2C	579	8	*L. major*	69.8	XP_001682225
**55**	PFR2C	77	1	*L. major*	69.8	XP_001682225
**18**	ribonucleoprotein p18	69	1	*L. major*	21.6	XP_001681946
**19**	ribonucleoprotein p18	177	3	*L. major*	21.6	XP_001681946
**20**	cytochrome c oxidase subunit V	273	3	*L. major*	22.4	XP_001684176
	Hypothetical protein	138	1	*L. major*	17.7	XP_001684176
**23**	Hypothetical protein	122	2	*L. major*	41.1	XP_001684940
**24**	Calmodulin	549	5	*T. cruzi*	16.8	XP_805243
**25**	Hypothetical protein	121	1	*L. major*	21.9	XP_001685804
**51**	ATPase beta subunit	717	7	*L. major*	56.5	XP_001683872

Supplementary Table 2
